# Neurogastroenterology and motility disorders in patients with cirrhosis

**DOI:** 10.1097/HC9.0000000000000622

**Published:** 2025-01-07

**Authors:** Francisco Idalsoaga, Gustavo Ayares, Hanna Blaney, Daniel Cabrera, Javier Chahuan, Hugo Monrroy, Ayah Matar, Houssam Halawi, Marco Arrese, Juan Pablo Arab, Luis Antonio Díaz

**Affiliations:** 1 Departamento De Gastroenterología, Escuela De Medicina, Pontificia Universidad Católica De Chile, Santiago, Chile; 2 Department of Medicine, Division of Gastroenterology, Schulich School of Medicine, Western University & London Health Sciences Centre, London, Ontario, Canada; 3 Universidad Finis Terrae, Escuela de Medicina, Facultad de Medicina, Universidad Fines Terrae, Santiago, Chile; 4 MedStar Georgetown University Hospital, Medstar Transplant Hepatology Institute, Washington, District of Columbia, USA; 5 Faculty of Medicine, Universidad de los Andes, Santiago, Chile; 6 Centro de Estudios e Investigación en Salud y Sociedad, Escuela de Medicina, Facultad de Ciencias Médicas, Universidad Bernardo O Higgins, Santiago, Chile; 7 Division of Gastroenterology and Hepatology, Mayo Clinic, Rochester, Minnesota, USA; 8 Department of Internal Medicine, Division of Gastroenterology, Hepatology, and Nutrition, Virginia Commonwealth University School of Medicine, Richmond, Virginia, USA; 9 MASLD Research Center, Division of Gastroenterology and Hepatology, University of California San Diego, San Diego, California, USA

**Keywords:** cirrhosis, gut-brain axis, liver cirrhosis, liver diseases, neurogastroenterology

## Abstract

Neurogastroenterology and motility disorders are complex gastrointestinal conditions that are prevalent worldwide, particularly affecting women and younger individuals. These conditions significantly impact the quality of life of people suffering from them. There is increasing evidence linking these disorders to cirrhosis, with a higher prevalence compared to the general population. However, the link between neurogastroenterology and motility disorders and cirrhosis remains unclear due to undefined mechanisms. In addition, managing these conditions in cirrhosis is often limited by the adverse effects of drugs commonly used for these disorders, presenting a significant clinical challenge in the routine management of patients with cirrhosis. This review delves into this connection, exploring potential pathophysiological links and clinical interventions between neurogastroenterology disorders and cirrhosis.

## Graphical abstract

**Figure:**
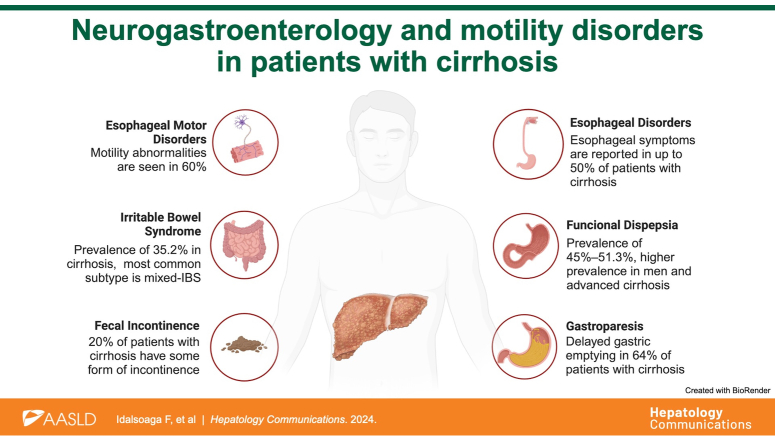

## INTRODUCTION

**FIGURE 1:**
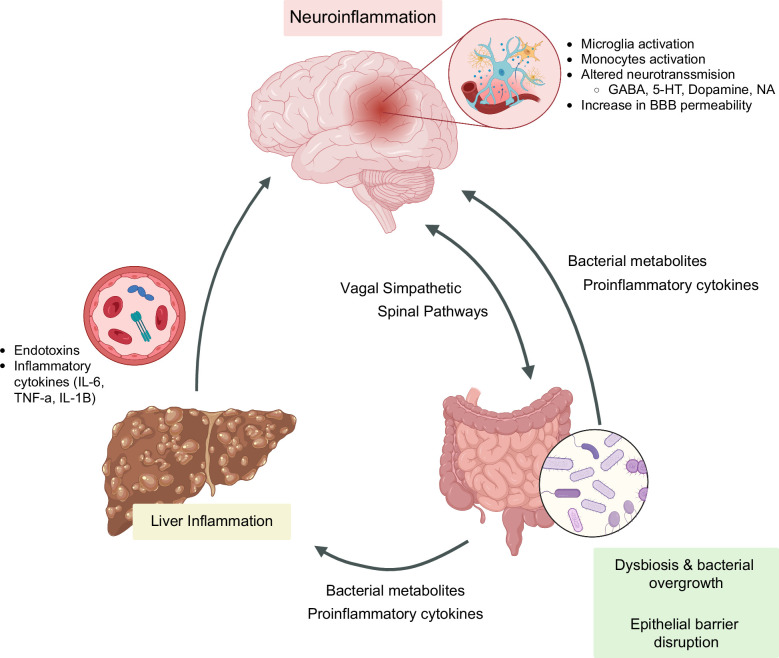
Gut-brain axis and its interactions with the liver. There is significant interaction between the brain and the gut through pathways mediated by neurotransmitters, bacterial metabolites, and associated cytokines, as well as the effect of the vagus nerve and spinal pathways. However, connections in the liver-gut axis have also been studied, mediated by dysbiosis, increased intestinal permeability, dysfunction of the immune system, and bacterial overgrowth. Abbreviations: 5-HT, 5-Hydroxytryptamine -Serotonin; BBB, blood-brain barrier; GABA, gamma-aminobutyric acid; NA, norepinephrine.

Neurogastroenterology and motility disorders are gastrointestinal conditions with complex pathophysiology, including visceral hypersensitivity, altered motility, impaired mucosal and immune function, changes in gut microbiota, and/or abnormal central nervous system processing of peripheral stimuli (Figure 1).^1^^,^^2^ These disorders of the gut-brain interaction (DGBI) are highly prevalent in the general population, affecting up to 40% of individuals worldwide.^3^ DGBI encompasses a broad spectrum of conditions, for which a detailed history and comprehensive physical examination are essential for establishing an accurate diagnosis. In addition, it is important to look for alarm symptoms and red-flag signs that may indicate an underlying organic or structural cause. The Rome IV criteria have defined the diagnostic standards for each entity (Table 1).^1^ These conditions have a high impact on individuals and significantly burden health care systems and societies.^6^^,^^7^ For instance, the annual cost of irritable bowel syndrome (IBS) per patient In the United States is estimated between $7423 and $7547.^6^ DGBI also leads to impaired quality of life, limited daily activities, sick leaves, decreased productivity, and reduced work performance.^8^^–^^10^

**TABLE 1:** Diagnostic criteria for the most common neurogastroenterology and motility disorders

Disorders	Diagnostic criteria
Functional heartburn^4^	Must include all the following^a^: (A) burning retrosternal discomfort or pain; (B) no symptom relief despite optimal antisecretory therapy; (C) absence of evidence that gastroesophageal reflux or eosinophilic esophagitis is the cause of the symptom; and (D) absence of major esophageal motor disorders.^b^
Reflux hypersensitivity^4^	Must include all the following^a^: (A) retrosternal symptoms including heartburn and chest pain; (B) normal endoscopy and absence of evidence that eosinophilic esophagitis is the cause of the symptoms; (C) absence of major esophageal motor disorders^b^; and (D) evidence of triggering of symptoms by reflux events despite normal acid exposure on pH- or pH-impedance monitoring.
Functional chest pain^4^	Must include all the following: (A) retrosternal chest pain or discomfort (cardiac causes should be ruled out); (B) absence of associated esophageal symptoms, such as heartburn and dysphagia; (C) absence of evidence that gastroesophageal reflux or eosinophilic esophagitis is the cause of the symptom; and (D) absence of major esophageal motor disorders.^b^
Functional dysphagia^4^	Must include all the following: (A) sense of solid and/or liquid foods sticking, lodging, or passing abnormally through the esophagus; (B) absence of evidence that esophageal mucosal or structural abnormality is the cause of the symptom; (C) absence of evidence that gastroesophageal reflux or eosinophilic esophagitis is the cause of the symptom; and (D) absence of major esophageal motor disorders.^b^
Globus^4^	Must include all the following: (A) persistent or intermittent, non-painful sensation of a lump or foreign body in the throat with no structural lesion identified on physical examination, laryngoscopy, or endoscopy; (B) occurrence of the sensation between meals; (C) absence of dysphagia or odynophagia; (D) absence of a gastric inlet patch in the proximal esophagus; (E) absence of evidence that gastroesophageal reflux or eosinophilic esophagitis is the cause of the symptom; and (F) absence of major esophageal motor disorders.^b^
Functional dyspepsia^4^	1. One or more of the following^d^: (A) bothersome postprandial fullness; (B) bothersome early satiation; (C) bothersome epigastric pain; and (D) bothersome epigastric burning. 2. AND: No evidence of structural disease (including at upper endoscopy) that is likely to explain the symptom.
Belching disorders^4^	Bothersome (ie, severe enough to impact usual activities) belching from the esophagus or stomach more than 3 d a week.
Rumination syndrome^4^	Must include all the following^d^: (A) persistent or recurrent regurgitation of recently ingested food into the mouth with subsequent spitting or remastication and swallowing; and (B) regurgitation is not preceded by retching.
Gastroparesis^5^	Gastric emptying scintigraphy demonstrating >60% retention at 2 h and >10% retention at 4 h for diagnosis.
IBS^4^	Recurrent abdominal pain on average at least 1 d/wk in the last 3 mo, associated with 2 or more of the following criteria: (A) related to defecation; (B) associated with a change in frequency of stool; and (C) associated with a change in form (appearance) of stool.
IBS-C^4^	Fulfill IBS criteria AND: >¼ (25%) of bowel movements with Bristol stool types 1 or 2 and <¼ (25%) of bowel movements with Bristol stool types 6 or 7.
IBS-D^4^	Fulfill IBS criteria AND: >¼ (25%) of bowel movements with Bristol stool types 6 or 7 and <¼ (25%) of bowel movements with Bristol stool types 1 or 2.
IBS-M^4^	Fulfill IBS criteria AND: >¼ (25%) of bowel movements with Bristol stool types 1 or 2 and >¼ (25%) of bowel movements with Bristol stool types 6 or 7.
IBS unclassified	Fulfill IBS criteria AND: bowel habits cannot be accurately categorized into 1 of the 3 groups (IBS-C, IBS-D, IBS-M).
Functional abdominal bloating/distension^4^	Must include both of the following^d^: (A) recurrent bloating and/or distension occurring on average at least 1 d/wk; abdominal bloating and/or distension predominates over other symptoms; and (B) there are insufficient criteria for a diagnosis of irritable bowel syndrome, functional constipation, functional diarrhea, or postprandial distress syndrome.
FC^4^	Must include 2 or more of the following^d^: (A) straining during more than ¼ (25%) of defecations; (B) lumpy or hard stools (Bristol Stool Form Scale 1-2) more than ¼ (25%) of defecations; (C) sensation of incomplete evacuation more than ¼ (25%) of defecations; (D) sensation of anorectal obstruction/blockage more than ¼ (25%) of defecations; (E) manual maneuvers to facilitate more than ¼ (25%) of defecations (eg, digital evacuation and support of the pelvic floor); (F) fewer than three spontaneous bowel movements per week; and (G) loose stools are rarely present without the use of laxatives (H) insufficient criteria for irritable bowel syndrome.
Dyssynergic defecation^4^	Inappropriate contraction of the pelvic floor as measured with anal surface EMG or manometry with adequate propulsive forces during attempted defecation.^d^
Fecal incontinence^4^	Recurrent uncontrolled passage of fecal material in an individual with a developmental age of at least 4 years.^e^
Centrally mediated abdominal pain syndrome^4^	Must include all the following^d^: (A) continuous or nearly continuous abdominal pain; (B) no or only occasional relationship of pain with physiological events (eg, eating, defecation, or menses)^c^; (C) pain limits some aspect of daily; (D) the pain is not feigned; and (E) pain is not explained by another structural or functional gastrointestinal disorder or other medical condition.

^a^ Criteria fulfilled for the last 3 months with symptom onset at least 6 months before diagnosis with a frequency of at least twice a week.

^b^ Achalasia/EGJ outflow obstruction, diffuse esophageal spasm, jackhammer esophagus, and absent peristalsis.

^c^ Criteria fulfilled for the last 3 months with symptom onset at least 6 months before diagnosis with a frequency of at least once a week.

^d^ Criteria fulfilled for the last 3 months with symptom onset at least 6 months before diagnosis.

^e^ Criterion fulfilled for the last 3 months.

Abbreviations: FC, functional constipation; IBS, irritable bowel syndrome; IBS-C, IBS predominant constipation; IBS-D, IBS with predominant diarrhea; IBS-M, IBS with mixed bowel habits.

**FIGURE 2:**
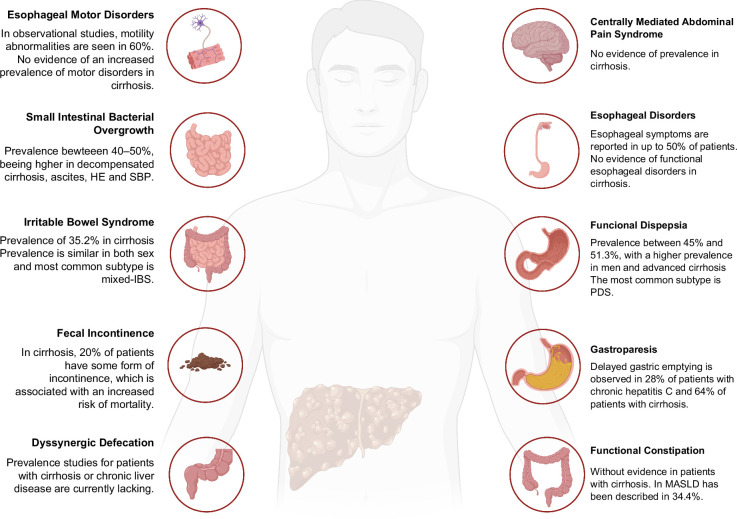
Prevalence and associations among different disorders in cirrhosis. Digestive pathologies are common in patients with cirrhosis, with many studies not establishing the relationship between the liver and the intestine. Abbreviations: IBS, irritable bowel syndrome; MASLD, metabolic dysfunction–associated steatotic liver disease; PDS, postprandial distress syndrome; SBP, spontaneous bacterial peritonitis.

Cirrhosis is also a relatively prevalent condition that imposes a significant burden on health care systems across the globe. Liver disease causes 2 million deaths annually and accounts for 4% of all deaths worldwide.^11^^,^^12^ Cirrhosis contributes significantly to the global burden of disability-adjusted life years, ranking as the 15th leading cause of disability-adjusted life years.^13^ Furthermore, it places a substantial strain on health care systems; in the United States alone, liver-related expenditure in 2016 was around $32.5 billion, with two-thirds of these costs attributed to inpatient and emergency department care.^14^ Many patients with cirrhosis also suffer from DGBI. In fact, several studies have described a significant association between DGBI and cirrhosis (Figure 2). However, the underlying pathophysiology of this association remains unclear and requires further investigation. Due to the high frequency of DGBI in individuals with cirrhosis, this review explores the association of chronic liver disease with neurogastroenterology and motility disorders, possible pathophysiological mechanisms behind this association, and potential clinical interventions and therapies.

## ESOPHAGEAL DISORDERS

Esophageal diseases and their associated symptoms are prevalent in the general population. An estimated 25% of people experience symptoms related to the esophagus, with heartburn being the most common (25%), followed by globus sensation (16%) and chest pain (13%).^15^^,^^16^ Functional esophageal disorders are characterized by typical esophageal symptoms (ie, heartburn, chest pain, dysphagia, and globus) that are not attributable to structural abnormalities, motor dysfunction, or gastroesophageal reflux disease (GERD).^1^^,^^17^ The global prevalence of Rome IV functional esophageal disorders is estimated at 6% in studies conducted through internet surveys.^3^ Esophageal symptoms are also very common in patients with cirrhosis. GERD symptoms are present in 55%–64% of cases, while 13% of patients experience dysphagia,^18^ especially those with esophageal varices or after procedures such as endoscopic band ligation.^19^ However, there is no published data on the prevalence of functional esophageal disorders in patients with cirrhosis.

According to the Rome IV classification, functional esophageal disorders include functional heartburn, reflux hypersensitivity, functional chest pain, functional dysphagia, and globus.^20^ The various functional esophageal disorders have clinical criteria (Table 1). The diagnosis of these disorders requires the exclusion of GERD, eosinophilic esophagitis, or major esophageal motor disorders. As such, it is often necessary to perform an upper gastrointestinal endoscopy and, in some cases, an ambulatory esophageal pH monitoring and/or an esophageal manometry. This presents a significant challenge in patients with cirrhosis due to the lack of evidence regarding the safety of performing pH-metry and manometry, particularly in patients with cirrhosis and esophageal varices, due to the potential risk of bleeding.^21^^,^^22^ In addition, esophageal biopsies to rule out eosinophilic esophagitis in patients with cirrhosis may carry a high risk of bleeding due to esophageal varices and thrombocytopenia.

The management of functional esophageal disorders is also a challenge in patients with cirrhosis. Diagnostic criteria for functional heartburn include the lack of symptom relief despite optimal antisecretory therapy, while proton pump inhibitors (PPIs) are the first-line treatment for functional chest pain. However, the safety of PPIs in patients with cirrhosis remains a controversial issue. PPIs have been associated with various side effects, including constipation, gastrointestinal infections, and HE, along with broader risks such as fractures, interstitial nephritis, anemia, and cognitive decline.^23^^–^^25^ Observational studies assessing the link between PPI use and HE have yielded inconsistent results. While some evidence indicates a dose-dependent increase in HE risk with PPIs, omeprazole and esomeprazole appear to have the lowest association with this complication.^24^ Long-term PPI use is also concerning due to the heightened risk of infections and decompensation in patients with cirrhosis,^26^ with reports even suggesting increased mortality in this population.^27^

The half-life of PPIs is prolonged in patients with cirrhosis, leading to persistent gastric acid suppression, which can promote bacterial overgrowth and translocation, potentially resulting in infections like spontaneous bacterial peritonitis (SBP).^26^ For instance, meta-analysis reported a 1.98-fold increased risk of bacterial infections due to PPIs.^28^ Thus, certain considerations are necessary when using PPIs in patients with cirrhosis. A review of 69 studies found that esomeprazole, omeprazole, and rabeprazole have no additional risks for patients with cirrhosis and Child-Pugh A or B, though dose reductions for omeprazole and rabeprazole are advised. In patients with Child-Pugh C cirrhosis, esomeprazole might be the only recommended PPI, with a maximum dose of 20 mg per day, as its risk profile remains similar to healthy individuals. Higher doses of esomeprazole (40 mg daily) lead to elevated plasma levels, and omeprazole shows increased exposure with more advanced cirrhosis.^24^ The use of pantoprazole and lansoprazole has been considered a potential risk in some reports due to the observed increase in plasma concentrations, up to 8-fold, across all stages of cirrhosis. However, further evidence is needed to clarify the actual risk associated with their use.^24^

For the management of functional chest pain, functional dysphagia, and globus, neuromodulators are the mainstay of treatment, with increasing evidence supporting their use. Although there is no direct evidence for some of these conditions, data from other DGBI are often extrapolated.^29^^,^^30^ Treatment options include tricyclic antidepressants (TCAs), selective serotonin reuptake inhibitors, serotonin-norepinephrine reuptake inhibitors, adenosine antagonists, trazodone, serotonin 5-hydroxytryptamine (5-HT) 3 receptor antagonists, 5-HT 4 receptor agonists, octreotide, gabapentin, pregabalin, and cannabinoid receptor agonists.^29^ The evidence on the safety of TCAs is mixed. TCAs are primarily metabolized by CYP450 enzymes, including CYP2D6, and have extensive protein binding, which could increase toxicity risk in patients with cirrhosis.^31^ Some reports link TCA use to sedation and a potential rise in HE; however, others have shown no significant drowsiness or encephalopathy when used short-term.^32^ The risk appears to be dose- and duration-dependent, so TCAs should be prescribed with caution.^32^ Another treatment option is psychological therapy, which has shown benefit^29^^,^^30^ and carries a low risk in patients with cirrhosis.

## ESOPHAGEAL MOTOR DISORDERS

Esophageal contractility is influenced by central and peripheral neural mechanisms. Vagal preganglionic motor nerves interact with postganglionic neurons in the myenteric plexus, which are crucial for regas a symptom in individuals with cirrhosis.ulating esophageal motility. The contraction amplitude is determined by a balance between excitatory cholinergic neurons and inhibitory nitric oxide producing neurons, with vagal efferent neurons playing a particularly significant role.^19^ Therefore, a reduction in parasympathetic activity may lead to decreased esophageal peristalsis.

Cirrhosis is associated with reduced parasympathetic activity, increased sympathetic activity, and heightened nitric oxide levels, potentially affecting esophageal motility and leading to diminished peristalsis.^33^^,^^34^ Alcohol consumption, a major contributor to liver disease, also impacts esophageal function. Studies in humans and animal models have shown that alcohol can decrease lower esophageal sphincter pressure and reduce contraction amplitude, further impairing mucosal clearance.^35^ A study in China involving 78 patients with cirrhosis found significant abnormalities in lower esophageal sphincter pressure and peristaltic characteristics compared to a control group.^36^ This mirrors findings from an Italian study.^37^ Notably, esophageal motility abnormalities are more pronounced in patients with esophageal varices, particularly large ones, and worsen with liver dysfunction.^38^ In addition, treatments for esophageal varices, such as endoscopic band ligation and endoscopic injection sclerotherapy, can temporarily alter motility, with endoscopic injection sclerotherapy having a more significant effect.^38^^–^^41^

Motor disorders, including achalasia and distal esophageal spasm, have been observed in patients with cirrhosis.^19^^,^^21^^,^^42^ Despite several studies, there is a lack of research using the Chicago Classification version 4.0 for diagnosing these disorders.^43^ A Brazilian study involving 74 patients with cirrhosis showed esophageal motility disorders in 60% of cases, primarily ineffective motility.^42^ Similarly, a Turkish study reported a 45.9% prevalence of motor disorders among patients with primary biliary cholangitis.^21^ While the precise mechanisms remain unclear, some studies suggest that cirrhosis may lead to autonomic neuropathy, contributing to these motility issues.^19^ Increased nitric oxide production and hormonal changes could also be factors.

For treating achalasia, pneumatic dilatation, laparoscopic Heller myotomy, or per-oral endoscopic myotomy (POEM) are recommended for Types I and II, while tailored myotomy approaches are suggested for Type III.^44^ Medical treatments, such as botulinum toxin injections or smooth muscle relaxants, are reserved for patients ineligible for definitive therapies.^44^ Limited evidence exists regarding these treatments in patients with cirrhosis. A small study from the United Kingdom evaluating various therapies in 14 patients with cirrhosis found effective symptom relief without complications.^45^ Similar case reports indicated successful outcomes with POEM in symptomatic patients with advanced cirrhosis.^46^^,^^47^ Some centers have proposed that performing POEM or laparoscopic Heller myotomy in patients with prior TIPS and variceal embolization may be a safer option.^48^^,^^49^ Given the limited data, a careful assessment of risks versus benefits is essential for patients with advanced cirrhosis.

## GASTRODUODENAL DISORDERS

Functional gastroduodenal disorders are very common in the general population. According to the Rome IV criteria, these include functional dyspepsia (FD), belching disorders, and rumination syndrome, with FD being by far the most frequent and the one with the most data in the general population.^50^ According to the Rome IV criteria, FD is defined by bothersome epigastric burning, epigastric pain, postprandial fullness, and early satiety (inability to finish a normal meal) with no structural disease (Table 1). A recent systematic review that included 44 studies, with 256,915 participants from 40 countries across 6 continents, reported a prevalence of 8.4% in the general population; however, when only studies using the Rome IV criteria were considered, this decreased to 6.8%.^50^ This systematic review also found that the prevalence of FD differs by country, economic status, geographical region, and sex, and the global prevalence has been gradually declining over the past decades. Three subtypes of FD are described: postprandial distress syndrome (PDS), epigastric pain syndrome (EPS), and overlapping PDS/EPS (Table 1). The most common subtype is PDS with a prevalence of 66.6%, followed by overlap PDS/EPS (18.1%) and EPS (15.3%).^3^^,^^51^

There is very limited evidence regarding the prevalence of FD in patients with cirrhosis, with only a few publications describing the prevalence of dyspepsia as a symptom in patients with cirrhosis. An observational study, which included 1506 participants with chronic viral hepatitis, found a prevalence of FD at 51.3%, with 49.9% having PDS and 46.7% EPS. In addition, the authors described a significant association between chronic hepatitis virus and the prevalence of FD, early satiety, postprandial distress, and epigastric pain.^52^ Moreover, the occurrence of FD is higher in males compared to females, with the onset typically manifesting approximately a decade later in this group. Interestingly, dyspeptic symptoms have been shown to progress in parallel with the liver disease stage.^53^

The medical management of FD has limited effectiveness, and no treatments are proven to modify its long-term natural history. The use of PPIs was examined in a systematic review of 25 RCTs among 8453 participants, concluding that PPIs are effective for FD treatment, regardless of dose or duration, compared to placebo (risk ratio: 0.88, 95% CI: 0.82–0.94). Moreover, PPIs may be slightly more effective than prokinetics for FD treatment (relative risk [RR]: 0.89, 95% CI: 0.81–0.99).^54^ Given the evidence supporting their benefit, it is reasonable to consider PPI use in these patients, while always weighing the potential risks in those with cirrhosis. In addition, *Helicobacter pylori* eradication has also shown a modest effect in treating dyspepsia, with an RR of symptom persistence at 0.90.^55^ Similarly, studies in patients with cirrhosis indicate that PPI treatment yields an RR of 0.88 for persistent symptoms compared to placebo.^56^

Low-dose TCAs have been suggested as a treatment for FD due to their peripheral pain-modifying effects, with drugs like amitriptyline, demonstrating benefits in FD. In a multicenter, randomized controlled study including participants without cirrhosis, amitriptyline demonstrated significant pain relief compared to placebo (OR: 3.1).^57^ In addition, in another randomized, placebo-controlled pilot trial involving 34 patients with FD with weight loss >10% without cirrhosis, mirtazapine significantly improved early satiation, quality of life, gastrointestinal-specific anxiety, nutrient tolerance, and weight loss in patients with FD.^58^ Both amitriptyline and mirtazapine have been linked to DILI, necessitating careful consideration.^59^^,^^60^ In particular, amitriptyline can cause sedation, constipation, and other anticholinergic effects, with anticholinergic medication use associated with increased overt HE in patients with cirrhosis^61^ (Table 2). Finally, randomized trials of rifaximin showed a significant increase in rates of adequate relief of global symptoms and postprandial fullness.^84^

**TABLE 2:** Pharmacological recommendations in patients with cirrhosis and neurogastroenterology and motility disorders

Disorders	Drug	Mechanism of action	Recommendations	Notable risks^a^
Functional heartburn	Amitriptyline^62^	TCA	Combine pharmacological interventions with usual lifestyle recommendations for heartburn. Reduces functional chest pain over a range from 18% to 67%.^63^	Dry mouth, sedation, drowsiness, constipation, dizziness, HE, and DILI (case reports).^64^^,^^65^ Main risk: QT prolongation.
	Fluoxetine^66^	SSRI	Modest effect on symptoms: Reduction of 37% vs. placebo 7%	Sedation and gastrointestinal disturbances. Main risk: worsening of hyponatremia.
Reflux hypersensitivity	Citalopram^67^	SSRI	Combine pharmacological interventions with usual lifestyle recommendations. Reduces 37% in pain thresholds.	Sedation and gastrointestinal disturbances, worsening of hyponatremia through SIADH.
	Baclofen^68^	GABA-B agonist that reduces TLESRs	Consider adjunctive agents, including alginate antacids, for breakthrough symptoms.	Common nausea, vomiting, constipation, and dyspepsia. Main risk: bradycardia and hypotension.
Functional chest pain	Sertraline and paroxetine^69^	SSRI	Combining low-dose amitriptyline with a PPI has been shown to be more effective than a double-dose PPI alone in refractory to standard PPI therapy patients.	Sedation and gastrointestinal disturbances. Main risk: worsening of hyponatremia.
Functional dysphagia	Trazodone^22^	Mixed agonist and antagonist of various serotonin receptors	Modest response rate (42%–49%), mostly effective on non-cardiac chest pain	Dry mouth, feeling faint, vomiting, and headache.^22^ More serious side effects may include suicide, mania, and arrhythmia.
	Ondansentron^22^	Serotonin 5-HT 3 receptor antagonist	Mostly improved the upper gastrointestinal symptoms of postprandial epigastric discomfort, flatulence, and heartburn.^70^ Consider ECG before initiating therapy.	Headaches, diarrhea, constipation (up to 9%), and dizziness.^71^ Main risk: DILI (rare), QT prolongation (rare).
	Cisapride, prucalopride^22^	5-HT 4 receptor agonists	Specific studies directly evaluating their efficacy in dysphagia are limited. Prokinetic agent. May enhance motility along the gastrointestinal tract.	Nausea, headache, diarrhea, and abdominal. Prucalopride is contraindicated in intestinal perforation or obstruction, Crohn’s disease, ulcerative colitis, and toxic megacolon.
	Gabapentin^22^	Binds to the α2δ subunit of voltage-gated calcium channels	Modest response rate (66%). Patients with encephalopathy might be contraindicated.	Dizziness, somnolence, and peripheral edema. Main risks: DRESS, anaphylaxis, angioedema, and respiratory depression (particularly when used with other CNS depressants).
	Pregabalin^22^	Similar to gabapentin it also reduces calcium influx at nerve terminals	As with gabapentin use in cirrhosis is not recommended in patients with HE.	
Globus	Gabapentin and amitriptyline, see above.			
Functional dyspepsia	Esomeprazole	PPI	CPS A or B up to 40 mg daily. CPS C up to 20 mg daily.	Infection, HE, constipation, diarrhea (up to 25% of patients with doses over 40 mg daily).^24^
	Omeprazole	PPI	Max dose 20 mg daily in all CPS.	Infection, HE, constipation, diarrhea (up to 13% of patients with doses over 40 mg daily).^24^
	Lansoprazole	PPI	Patients with moderate to severe hepatic impairment should be kept under regular supervision and a 50% reduction of the daily dose is recommended.	High risk of exposure to acid suppression and anaphylactic reaction. Case reports: DRESS syndrome.^24^
	Pantoprazole	PPI	In patients with severe hepatic impairment, the maximum daily dose of pantoprazole is 20 mg. Liver enzymes should be monitored regularly, and treatment should be discontinued if enzyme levels increase.	High risk of exposure to acid suppression. Case reports: DILI.^24^
	Buspirone and Tandospirone	5-HT receptors agonist, fundus Relaxation	Small studies show significant improvement in gastric accommodation, gastric emptying of liquids, and dyspeptic symptoms in patients with functional dyspepsia. Can slow gastric emptying at 20 mg.	Nausea and drowsiness if liver insufficiency is up to 12%.^64^
	Amitriptyline see above.			
	Mirtazapine	TCA	Low level of evidence	Case reports: DILI.^72^
Belching disorders	Baclofen, see above			
Rumination syndrome	Baclofen and TCA, see above			
Gastroparesis	Metoclopramide	Dopamine D2 receptor antagonist, and is also a mixed 5-HT3 receptor antagonist/5-HT4 receptor agonist	Increased gastric emptying by enhancing antral contractions as well as decreasing postprandial fundus relaxation. Prokinetic properties of metoclopramide are limited to the proximal gut.^73^	High risk of extrapyramidal side effects and potential irreversible tardive dyskinesia in chronic use (1%–10%; however, this may be overreported).^74^
	Domperidone	Dopamine D2 receptor antagonist	May be considered	QT prolongation up to 5%.^75^
	Ondansetron, see above.			
IBS-D	Loperamide	Synthetic opioid	Up to 16 mg in severe cases	Drowsiness, vomiting, abdominal pain, burning, and rrhythmia. Higher risk in higher liver failure (1.9%–3.2%).^71^^,^^76^
	Mebeverine	Antispasmodic	There is no evidence of cirrhosis. It may be considered with caution.	Constipation.
	Otilonium Bromide	Antispasmodic	There is no evidence of cirrhosis. It may be considered with caution.	Constipation.
	Dicyclomine^77^	Antispasmodic	There is no evidence of cirrhosis. It may be considered with caution.	Dry mouth, dizziness, and blurred vision.
	Hyoscine (scopolamine)^77^	Antispasmodic	There is no evidence of cirrhosis. It may be considered with caution.	Dry mouth and blurred vision.
	Amitriptyline, see above.			
	Low-FODMAPs diet	Diet	It may be considered with caution.	No significant adverse effects reported.
IBS-C	Kiwi	Diet	Without known adverse effects of cirrhosis	No significant adverse effects reported.
	Polietilenglicol 3350 (PEG)	Linear polymer Osmotic laxative	Titrate in conjunction with EH treatment	No significant adverse effects reported.^78^
	Lactulose 33%	Nonabsorbable sugar	PEG is more effective for symptom control.	Bloating.^79^^,^^80^
	Plecanatide^81^	Guanylate cyclase-C agonist	There is no evidence of cirrhosis. It may be considered with caution.	Diarrhea.
	Tenapanor^82^	Inhibitor of the gastrointestinal sodium/hydrogen exchanger isoform 3	There is no evidence of cirrhosis. It may be considered with caution.	Diarrhea.
Functional abdominal bloating/distension	Low-FODMAPs diet, see above. Antidepressants (TCAs, SSRI, and SNRIs), see above. Prosecretory and promotility agents (lubiprostone, prucalopride, linaclotide), see above.			
	Probiotics (*Bifidobacterium infantis, Bifidobacterium lactis*)	Modifications in intestinal microbiota	Without known adverse effects in cirrhosis, caution is advised in its use for patients with immunosuppression.	Sepsis, bacteremia, fungemia, and endocarditis in immunocompromised patients.
Fecal incontinence	Psyllium, dietary fiber	Stool bulking	In some studies no differences between the loperamide and psyllium groups.^83^	Bloating in 3.2%.^79^
	Biofeedback therapy	Mind-body technique	No significant risks.	No significant adverse effects reported.
	Low-FODMAPs diet, see above.			
DD	Biofeedback therapy, see above.			
Centrally mediated abdominal pain syndrome	TCAs, SSRI, and SNRIs, see above.			

*Note*: There is considerable overlap in treatment options in different pathologies.

^a^ Related to cirrhosis.

Abbreviations: 5-HT, 5-hydroxytryptamine; CPS, Child-Pugh score; DRESS, drug reaction with eosinophilia and systemic symptoms; ECG, electrocardiogram; FD, functional dyspepsia; FI, fecal incontinence; FODMAPS, fermentable oligo-, di-, monosaccharides and polyols; GERD, gastroesophageal reflux disease; IBS-C, irritable bowel syndrome constipation predominantly; IBS-D, irritable bowel syndrome diarrhea predominantly; PPI, proton pump inhibitor; SIAD, syndrome of inappropriate antidiuretic hormone secretion; SNRI, serotonin-norepinephrine reuptake inhibitor; SSRI, selective serotonin reuptake inhibitor; TCA, tricyclic antidepressant; TLESR, transient lower esophageal sphincter relaxation.

There is a lack of evidence regarding belching disorders in patients with liver disease, with no specific publications involving patients with cirrhosis. Belching disorders, according to Rome IV, can be classified as excessive supragastric belching (from the esophagus) or excessive gastric belching (from the stomach), depending on the clinical characteristics (Table 1). A Japanese survey-based study in the general population involving 10,000 adults reported a 1.5% prevalence of belching disorders. The study also identified increased ORs associated with several factors, including reflux esophagitis, thyroid disease, GERD, FD, feeling full after eating, and small or large number of mastication.^85^ In cirrhosis, belching symptoms have been reported, though these symptoms do not necessarily fulfill the Rome IV criteria for belching disorders, in up to 18.7% of patients. These symptoms are associated with liver disease severity, the use of lactulose, the presence of ascites, and psychological distress.^86^^,^^87^ The use of coffee has also been associated with a higher frequency of belching disorders.^88^ The cornerstone of treatment for both subtypes is a comprehensive clarification of the etiology of these symptoms, making the patient aware that this is a behavioral disorder. In this sense, speech therapy,^89^ abdominal breathing,^90^ and cognitive behavioral therapy^91^ are the mainstays of therapy. All these treatments are safe for patients with cirrhosis and are likely helpful in managing symptoms in these patients. In patients with decompensated cirrhosis, optimization of treatment of ascites and the use of other laxatives than lactulose, such as polyethylene glycol 3350, may also be used in the management of belching symptoms.

Regarding rumination syndrome, a multicenter study that surveyed 54,127 subjects found an overall prevalence of 3.1%. Associated factors included depression (OR: 1.46), anxiety (OR: 1.8), body mass index (OR: 1.04), and female sex (OR: 1.19), with the highest risk observed in subjects with 4 gastrointestinal regions affected by DGBI (OR 15.9).^92^ However, there are no reports on the prevalence of rumination syndrome or rumination as a symptom in individuals with cirrhosis. As with belching disorders, the cornerstone of therapy is behavioral therapy.^93^ Another treatment that has been studied is baclofen; a randomized double-blinded placebo-controlled study on 25 patients with supragastric belching and rumination syndrome who did not respond to PPIs found that baclofen reduced the number of rumination episodes.^94^ Bacofen therapy could be considered in cirrhosis, with baclofen having a favorable safety profile in patients with liver disease, with these data primarily derived from managing alcohol use disorder in individuals with advanced alcohol-associated liver disease.^95^

## GASTRODUODENAL MOTOR DISORDERS

Gastroparesis is a motility disorder that is characterized by delayed gastric emptying in the absence of physical obstruction or blockage (Table 1).^96^ Symptoms are usually chronic and can lead to extraintestinal complications and even hospitalization. The global burden of disease is significant, with gastroparesis affecting 268 per 100,000 US adults.^97^ The number of gastroparesis-related hospitalizations has increased, adding to the economic burden of the disease.^98^ The most common underlying etiologies of gastroparesis include diabetes mellitus and postsurgical gastroparesis; however, many cases are idiopathic with some association with autoimmunity. Moreover, histopathological assays show morphological defects in smooth muscle, Cajal interstitial cells, and enteric neurons along with high levels of inflammatory cells and falling levels of neurotransmitters.^99^

Gastric emptying studies of gastroparesis in patients with cirrhosis using solid meal scintigraphy^100^^,^^101^ found that 28% of patients with chronic hepatitis and 64% of patients with cirrhosis experienced delayed gastric emptying. Incidence of gastroparesis is higher in patients with cirrhosis, prompting the consideration of a potential role of delayed gastric emptying in relation to liver function and portal hypertension.^101^ A study that included patients with HCV infection with and without cirrhosis (controls) found that compared to controls, patients with cirrhosis undergoing solid phage gastric emptying scintigraphy had higher abnormal gastric retention (70.7% vs. 26.1%; *p* < 0.001). Moreover, the study revealed a positive correlation between the presence of autonomic dysfunction and underlying motility disorders, such as delayed gastric emptying, suggesting that autonomic dysfunction might play a role in the pathogenesis of gastroparesis in patients with cirrhosis.^100^ Patients with cirrhosis also had high levels of postprandial insulin, glucose, and glucagon-like peptide 1 with lower levels of ghrelin. This hormonal imbalance could partially explain gastroparesis in patients with cirrhosis.^102^ In addition, many people with metabolic dysfunction–associated steatotic liver disease (MASLD) have long-term diabetes and may be at increased risk of gastroparesis. Overall, cirrhosis has been understudied as a potential cause of gastroparesis and other gastroduodenal conditions,^103^ and further research is needed. One area of future research that would be particularly useful is the relation between gastroparesis and clinically significant portal hypertension with ascites.

Prokinetic drugs might be beneficial in reducing the recurrence of symptoms in patients with FD and evidence of gastroparesis (RR: 0.81).^56^^,^^104^ However, there are significant risks of adverse effects in the overall population, including the risk of extrapyramidal complications such as irreversible tardive dyskinesia which can be seen with chronic use of metoclopramide. Similarly, domperidone can cause cardiac arrhythmias, and ondansetron (an antiemetic) can cause liver enzyme abnormalities.^105^ In a study comparing metoclopramide and domperidone in patients with cirrhosis and ascites, the use of metoclopramide was associated with a significantly reduced urinary output and urinary sodium,^106^ whereas domperidone exhibited a better safety profile and a lower rate of adverse effects. In this sense, domperidone should be the preferred choice in patients with cirrhosis requiring prokinetic therapy (Table 2). More research is needed to study the safety and efficacy of other prokinetics, including prucalopride, in patients with cirrhosis. For example, prucalopride both enhances gastric emptying in gastroparesis and improves colonic transit, which may, in theory, have a role in the management of HE in patients with cirrhosis and impaired motility.

In patients with gastroparesis who are refractory to medical therapy and have moderate to severe symptoms, particularly nausea and vomiting, gastric POEM should be considered. This procedure may be an option for patients with gastroparesis and cirrhosis; however, further studies are needed to assess its safety in this population.^105^^,^^107^ Finally, buspirone and tandospirone are 5-HT receptor agonists that enhance fundal relaxation; however, evidence for their effectiveness is low. Compared with healthy individuals, these drugs can have higher bioavailability in patients with liver disease, which may increase the risk of adverse effects such as nausea and drowsiness (Table 2).^64^^,^^84^^,^^108^

## BOWEL DISORDERS

Bowel symptoms are extremely common in the general population and are particularly common in patients with cirrhosis. Up to 80% of patients with cirrhosis report digestive symptoms attributable to bowel disorders, with abdominal bloating being the most frequently reported, present in ~49.5% of patients, followed by abdominal pain in 24%.^109^ The most common bowel disorders classified in the Rome IV criteria are IBS, functional constipation, and functional abdominal bloating/distension.^1^

IBS is a complex pathophysiological disease characterized by recurrent abdominal pain with specific criteria (Table 1).^110^^,^^111^ The global prevalence of IBS in the general population is ~11.2%^112^; however, it varies significantly, ranging from 1.1% in countries like France and Iran to 35.5% in Mexico.^113^^,^^114^ This condition is more frequent in young and female individuals,^115^ and it tends to be associated with other DGBI. IBS has a significant impact on quality of life and health care delivery, generating substantial economic costs globally.^113^^,^^116^ IBS is classified into 4 subtypes according to the Rome IV criteria: IBS with constipation-predominant (IBS-C), IBS with diarrhea-predominant (IBS-D), IBS with mixed bowel habits, and IBS unclassified, with IBS-C and IBS-D being the most common subtypes.^117^

In patients with chronic liver disease, a higher prevalence of IBS has been reported. In patients with MASLD, the prevalence of IBS is 35.3%, and IBS is 3 times more likely to coexist with MASLD.^118^ Among those with MASLD and IBS, the most common subtypes are mixed-IBS (48%), diarrhea-predominant IBS (28%), and constipation-predominant IBS (18%), with 38% experiencing severe symptoms as assessed using the IBS Severity Scoring System questionnaire.^119^ Furthermore, individuals with MASLD appear to have more risk and associated factors for IBS, such as overlapping nutritional and dietary factors, changes in the gut microbiome, gut permeability, immunity, small intestinal bacterial overgrowth (SIBO), and bile acid metabolism.^120^ Patients with chronic HCV infection higher prevalence of IBS, particularly the constipation-predominant subtype.^52^

The management of IBS, regardless of its subtype, requires an integrated approach. This includes an effective patient-provider relationship, education, dietary modifications, pharmacotherapy, and behavioral and psychological treatment.^121^ Interestingly, among patients with symptoms suggestive of IBS, only a percentage (50%) receive medical attention and therapy.^122^ Nutritional management is an important topic, as one of the most commonly associated factors with the exacerbation of symptoms among patients with IBS is food ingestion.^123^ Although patients with IBS often incriminate specific food items, only 11%–27% of these foods are correctly identified with formal, blinded food challenge studies.^123^ Regarding nutritional management, numerous diets have been proposed for IBS management. In this context, a diet low in fermentable oligo-, di-, monosaccharides, and polyols (FODMAPs) has demonstrated beneficial effects on IBS symptoms in randomized controlled trials.^124^ Therefore, guidelines show a strong recommendation, albeit with a low level of evidence, for the use of a low FODMAPs diet, as well as gut-directed psychological therapies, which have a low risk of adverse effects.^125^ In patients with cirrhosis, dietary restrictions or changes should be implemented with caution, as many patients with cirrhosis have protein-calorie malnutrition.^126^ Sarcopenia affects approximately one-third of patients with cirrhosis, with prevalence exceeding 50% among those with alcohol-associated liver disease or with Child-Pugh C cirrhosis. In addition, sarcopenia has a significant impact on the mortality of patients with cirrhosis, independently associated with approximately a 2-fold higher risk of mortality. Moreover, mortality rates tend to increase with the severity or duration of sarcopenia.^127^^–^^129^ Although there are no studies specifically addressing the effect of a low FODMAPs diet on sarcopenia in patients with cirrhosis, research on older adults, another population with significant sarcopenia and mortality association, suggests that a low FODMAPs diet, when supervised by experienced dietitians, is clinically effective without compromising nutritional intake.^130^ In this context, it has been proposed that a low FODMAPs “gentle diet” should be considered to minimize dietary restrictions and simplify dietary changes.^130^ This gentle approach seems reasonable in patients with cirrhosis, with careful consideration of significant restrictions and special attention to those that may exacerbate sarcopenia. Therefore, it is crucial that such a diet be implemented under the supervision of qualified personnel to guide an initial phase of dietary restriction (typically lasting only 2–6 wk), followed by reintroduction and then personalization of the foods that need to be restricted.

Treating IBS with pharmacological therapy depends on the patient’s primary symptoms and the subtype of IBS. To manage abdominal pain, the use of antispasmodic agents or tricylic antidepressents is recommended. In IBS, pain is mediated by central and peripheral mechanisms, leading to smooth muscle spasms that trigger pain. In this context, the use of otilonium bromide, mebeverine, dicyclomine, and hyoscine has demonstrated benefits in pain relief and is recommended in different clinical guidelines.^77^^,^^125^^,^^131^^,^^132^ However, there is no evidence regarding the safety of these medications in cirrhosis. The potential risk of their use in cirrhosis, due to their antimuscarinic effects, lies in the generation of constipation from their strong inhibition of intraluminal fluid secretion, which could exacerbate HE. Another medication with evidence for pain management is peppermint oil, which also inhibits smooth muscle contraction. A randomized, double-blind, placebo-controlled clinical trial involving 72 patients showed a 40% reduction in pain scores when using peppermint oil compared to placebo.^133^ Peppermint oil has not been studied in patients with cirrhosis, though has a favorable safetly profile.

In patients with IBS-D, synthetic opioids that primarily affect opiate receptors in the intestine are the first-line treatment to treat diarrhea. Among these, loperamide is the most commonly used, as it does not cross the blood-brain barrier and has fewer central adverse effects. However, in patients with cirrhosis, it should be used with caution due to the risk of inciting HE. It has not been linked to serum liver enzyme elevations during therapy or to clinically apparent liver injury. The use of 5-HT3 receptor antagonists, such as ramosetron^134^ and ondansetron,^135^ represents additional therapeutic options for this group of patients. These medications are likely to have a better safety profile in cirrhosis, but they can also produce constipation.

As a second-line therapy for pain management in IBS-D, the use of low-dose TCAs has gained importance in recent years. A recent randomized, double-blind, placebo-controlled trial that included 463 participants demonstrated a clear benefit in the IBS Severity Scoring System, proving to be a good and safe option for patients with IBS.^136^ However, the use of low-dose TCAs (primarily amitriptyline) is associated with adverse effects in cirrhosis, such as sedation, constipation, and other anticholinergic effects,^59^^–^^61^ which could be harmful by increasing the risk of HE.

In patients with IBS-C, the use of osmotic laxatives are helpful in treating constipation. Polyethylene glycol is a safe and effective option.^137^^,^^138^ However, lactulose is often not recommended due to its tendency to worsen bloating and discomfort, which may lead to poor adherence. Despite this, it may be a viable treatment option in cirrhosis due to its benefits for HE.^139^ The use of kiwi fruit has also been reported to improve defecation frequency and even alleviate pain, though these studies did not include patients with cirrhosis.^140^^–^^142^

As second-line therapies for IBS-C, linaclotide may be utilized.^143^^–^^145^ This medication has a dual action: it increases intraluminal fluid secretion, provides a laxative effect, and modulates colonic nociceptors, thereby offering analgesic benefits. Linaclotide has been shown to reduce abdominal pain, bloating, and bowel symptoms. Similarly, lubiprostone has been evidenced to improve global intestinal symptoms in IBS-C.^146^ In addition, 5-HT4 receptor agonists, such as prucalopride, promote gut motility by activating serotonergic pathways.^147^ Another beneficial medication is plecanatide, which stimulates the guanylate cyclase-C receptor and has been shown to significantly improve both abdominal pain and constipation symptoms of IBS-C, with a favorable safety profile.^81^ Finally, tenapanor, an inhibitor of the gastrointestinal sodium-hydrogen exchanger-3, has demonstrated improvements in abdominal and global IBS-C symptoms.^82^ However, limited safety data exist for these medications in patients with cirrhosis. Given the potential for adverse effects, they should be used cautiously in this population, with close monitoring for side effects and careful clinical assessment. Due to the risk of diarrhea affecting fluid balance and electrolyte levels, laxatives should be used cautiously in patients with decompensated cirrhosis to prevent acute kidney injury or HE.

Abdominal bloating/distension is the primary gastrointestinal symptom reported by patients with cirrhosis, with up to 49.5% of individuals experiencing this symptom.^109^ It is likely multifactorial in origin and has been associated with liver disease severity, frequent use of lactulose, and the presence of ascites. These factors represent significant risk elements that often coexist in patients with cirrhosis.^87^^,^^148^ Although functional abdominal bloating/distension as a distinct entity has not been directly studied in this population, sometimes it is necessary to consider and rule out the presence of SIBO when addressing these symptoms. The prevalence of SIBO is high in patients with cirrhosis.^149^^–^^154^ SIBO is characterized by the presence of increased numbers of bacteria in the small intestine (Table 1).^152^ SIBO has been associated with motility disorders of small intestine, scleroderma, diabetes mellitus, Billroth II gastrojejunostomy, IBS, and intestinal obstructions after strictures.^152^ Although a spectrum, symptoms include flatulence, bloating, abdominal pain, and diarrhea.^155^ Patients with cirrhosis have a prevalence of SIBO of up to 50%, determined by glucose or lactulose hydrogen breath test.^149^^–^^151^ In addition, the prevalence of SIBO increases with the severity of cirrhosis^156^ particularly with serum bilirubin ≥2 mg/dL and ascites.^152^ In a systematic review and meta-analysis comprising 21 studies, the prevalence of SIBO in cirrhosis was found to be 40.8% and was notably higher in patients with decompensated cirrhosis and in patients presenting with ascites, minimal HE, bacterial translocation, SBP, and prolonged orocecal transit time. Importantly, it was not found to be associated with hypocoagulation.^61^ In MASLD, SIBO prevalence has been estimated at 35%, with male sex considered as a risk factor as well as alcohol consumption.^151^^,^^154^

Risk factors for SIBO in patients with cirrhosis may result from impaired intestinal motility and delayed transit time and worsen with more severe liver disease. Another significant factor is the abnormal activity of the migrating motor complex in patients with cirrhosis due to portal hypertension.^51^ In patients with cirrhosis, the presence of SIBO can have profound clinical consequences. The increased intestinal permeability predisposes bacterial translocation into the systemic circulation, confirmed by bacterial DNA analysis, suggesting that SIBO could be a major risk factor for bacterial translocation and SBP, particularly in patients with ascites.^150^ Antibiotics are the mainstay of treatment; however, due to their high risk of DILI, their use must be carefully evaluated.^157^ As such, nonabsorbed antimicrobial therapies such as rifaximin, or with low liver toxicity such as ciprofloxacin or norfloxacin should be preferentially used in these patients.^158^ These antibiotics are commonly used in patients with cirrhosis as a primary or secondary prophylaxis against SBP (and for HE in the case of rifaximin). Given that these antibiotics are frequently used in patients with cirrhosis, further research is needed to evaluate their use in treating SIBO in this population in the context of increasing resistance to antibiotics.

Regarding the treatment of functional bloating, once SIBO has been excluded, therapeutic options include dietary changes (low FODMAPs diet under the care of a dietician), probiotics, antibiotics, prokinetic agents, antispasmodics, neuromodulators, and biofeedback.^159^ Each of these has a distinct safety profile in patients with cirrhosis, as outlined in this review.

Functional constipation is characterized by irregular bowel movements, also with specific Rome IV criteria (Table 1). The pathophysiology is not entirely understood and includes altered central perception, abnormalities in motor function, visceral hypersensitivity, and delayed colonic transit.^160^ The global prevalence of functional constipation is 14% in the overall population^161^ and up to 34% in patients with MASLD.^119^ The main treatment for this condition is laxatives, such as polyethylene glycol and lactulose, which are safe to use in cirrhosis and a pillar of treatment in cirrhosis. However, the most effective laxative for functional constipation and cirrhosis has not been determined.^125^

## ANORECTAL DISORDERS

Anorectal disorders can have a significant impact on quality of life.^162^ Functional anorectal disorders include fecal incontinence (FI), disorders associated with functional anorectal pain (such as levator ani syndrome, unspecified functional anorectal pain, and proctalgia fugax), and those related to defecation disorders.^1^

FI is the involuntary loss of liquid or solid stool (Table 1) and is one of the most common anorectal disorders. Its prevalence ranges from 2.2% to 15% in the general population but can reach up to 46% in nursing homes.^163^ FI has numerous risk factors such as physical limitations, poor general health, female sex, age, urgency, loose stools, obesity, surgery, perianal injury by dysfunctional rectal sensorimotor activity, pudendal neuropathy, and anal sphincter weakness.^164^^,^^165^ There is a lack of data on the prevalence and incidence of FI in patients with cirrhosis. However, one-fifth of patients with cirrhosis report some form of incontinence, either fecal or urinary,^166^ and evidence suggests that FI negatively impacts quality of life and is associated with an increased risk of mortality.^166^ The exact cause of this association is unclear, though it may be due to an overlap of multiple health issues. In this context, sarcopenia may play a role. Sarcopenia and frailty are highly prevalent in cirrhosis and other chronic liver conditions and are linked to muscle loss and functional disabilities over time, potentially contributing to the presence of FI.^129^^,^^167^^,^^168^ In addition, many patients with cirrhosis are on lactulose, an osmotic laxative that can increase the risk of fecal urgency and FI. Finally, anorectal varices, which affect up to 89% of patients with cirrhosis and portal hypertension, may also play a potential role in increasing the risk of FI in patients with cirrhosis.^169^

Among the disorders associated with anorectal pain, the most common is proctalgia fugax. This condition is characterized by hyperacute anal pain, affecting between 8% and 18% of the general population, with similar prevalence in men and women.^170^^,^^171^ It is typically a benign disorder; for most patients, episodes of pain are brief and infrequent enough that reassurance and explanation are sufficient. However, in elderly patients with more intense symptoms, treatment may be necessary, which can include the inhalation of salbutamol, clonidine, amyl nitrite, or nitroglycerin.^172^ There is no data on the prevalence or safety of these treatments in patients with cirrhosis; however, the use of inhalational therapy does not pose a greater risk in individuals with cirrhosis.

Regarding the treatment of FI, some studies suggest that education regarding FI may be effective in treatment along with a low FODMAPs diet.^170^ Products that have a stool “bulking” effect and fiber supplementation, especially psyllium, may help reduce FI episodes.^171^^–^^173^ Some evidence suggests that gum Arabic can help reduce FI episodes.^171^ More evidence is needed to draw definitive conclusions on which products are superior in treating FI. As these products are nonabsorbent, the main concern with their use is tolerance, as many of them lead to increased bloating (Table 2), a side effect that is often poorly tolerated by patients with cirrhosis.

Among defecation disorders, dyssynergic defecation (DD) is a lack of coordination between the pelvic floor and abdominal muscles, leading to ineffective rectal and abdominal pushing forces and resulting in defecation difficulties (Table 1).^173^ DD has been found in 30%–50% of patients with chronic constipation.^174^^,^^175^ Some studies have suggested that DD can be induced by cirrhosis as it affects gastrointestinal motility.^176^^,^^177^ Over the last 2 decades, biofeedback therapy using visual and verbal feedback techniques has emerged as a useful option in patients with DD.^174^^,^^178^ Biofeedback therapy is safe in patients with cirrhosis and should be considered over pharmacological options.^179^ However, a limitation of biofeedback is the need for patient cooperation, which may be limited in a patient with cirrhosis and HE. There is also evidence supporting this therapy in FI, where it could be a low-risk option.^180^^,^^181^

## CENTRALLY MEDIATED ABDOMINAL PAIN SYNDROME

Centrally mediated abdominal pain syndrome, formerly known as functional abdominal pain syndrome, results from central sensitization with disinhibition of pain signals, rather than increased peripheral afferent excitability. This condition is relatively rare, with a prevalence of 0.5%–2.1%.^182^ It is characterized by continuous or nearly continuous abdominal pain, minimal or no relationship of pain with physiological events, limitations on daily functioning, and the absence of explanation by any structural or functional gastrointestinal disorder or other medical condition.^1^ Management of centrally mediated abdominal pain syndrome can be challenging, emphasizing the importance of establishing an effective patient-physician relationship.^182^ Therapeutic options include TCAs, selective serotonin reuptake inhibitors, and serotonin-norepinephrine reuptake inhibitors, often in conjunction with psychological treatment. Although there is no evidence of prevalence or association with cirrhosis for this entity, precautions regarding the use of neuromodulators should be observed due to the potential risk of precipitating HE.^59^^,^^60^

## CONCLUSIONS

Neurogastroenterology and motility disorders are common conditions in patients with cirrhosis and can have a significant impact on the quality of life in these individuals. There is limited evidence regarding the epidemiology and the pathophysiology of these conditions in patients with cirrhosis. Moreover, the medical therapies that are often used to treat these conditions can carry additional potential risks in patients with cirrhosis, with special consideration needed before their use in this patient population. High-quality studies are needed to assess the epidemiology and therapeutic options for these disorders in patients with cirrhosis.
